# Analysis of *Polygala tenuifolia* Transcriptome and Description of Secondary Metabolite Biosynthetic Pathways by Illumina Sequencing

**DOI:** 10.1155/2015/782635

**Published:** 2015-10-12

**Authors:** Hongling Tian, Xiaoshuang Xu, Fusheng Zhang, Yaoqin Wang, Shuhong Guo, Xuemei Qin, Guanhua Du

**Affiliations:** ^1^Research Institute of Economics Crop, Shanxi Academy of Agriculture Science, Fenyang, Shanxi 032200, China; ^2^Modern Research Center for Traditional Chinese Medicine, Shanxi University, No. 92 Wucheng Road, Taiyuan, Shanxi 030006, China; ^3^College of Chemistry and Chemical Engineering, Shanxi University, No. 92 Wucheng Road, Taiyuan, Shanxi 030006, China; ^4^Institute of Materia Medica, Chinese Academy of Medical Sciences, Beijing 100050, China

## Abstract

Radix polygalae, the dried roots of *Polygala tenuifolia* and *P. sibirica*, is one of the most well-known traditional Chinese medicinal plants. Radix polygalae contains various saponins, xanthones, and oligosaccharide esters and these compounds are responsible for several pharmacological properties. To provide basic breeding information, enhance molecular biological analysis, and determine secondary metabolite biosynthetic pathways of *P. tenuifolia*, we applied Illumina sequencing technology and de novo assembly. We also applied this technique to gain an overview of *P. tenuifolia *transcriptome from samples with different years. Using Illumina sequencing, approximately 67.2% of unique sequences were annotated by basic local alignment search tool similarity searches against public sequence databases. We classified the annotated unigenes by using Nr, Nt, GO, COG, and KEGG databases compared with NCBI. We also obtained many candidates CYP450s and UGTs by the analysis of genes in the secondary metabolite biosynthetic pathways, including putative terpenoid backbone and phenylpropanoid biosynthesis pathway. With this transcriptome sequencing, future genetic and genomics studies related to the molecular mechanisms associated with the chemical composition of *P. tenuifolia* may be improved. Genes involved in the enrichment of secondary metabolite biosynthesis-related pathways could enhance the potential applications of *P. tenuifolia *in pharmaceutical industries.

## 1. Introduction

As a part of commonly known traditional Chinese medicinal plants, Radix polygalae (RP) can be obtained from* Polygala tenuifolia* Willd. and* P. sibirica *L., which are listed in the Chinese Pharmacopoeia (2010); RP elicits sedative, antipsychotic, expectorant, and anti-inflammatory effects. High consumption and decreased regeneration of wild resources of RP have prompted researchers to domesticate and cultivate it and a large-scale planting has been performed since early 1980s. Thus far, RP from* P. tenuifolia* has been the most commonly used variety, so* P. tenuifolia *was used in this study.* P. tenuifolia* is a perennial herb mainly distributed in northeast, north, and northwest of China. This herb contains various medicinal components, such as saponins, xanthones, and oligosaccharide esters, which exhibit tonic, sedative, antipsychotic, expectorant, and other pharmacological effects [1]. In previous studies, chemical and pharmacological properties of triterpenoid saponins, one of the most important medicinal components of* P. tenuifolia*, were investigated [2]. However, studies on the biosynthetic pathway of triterpenoid saponins in* P. tenuifolia* have been rarely conducted compared with those in* licorice*,* ginseng*,* notoginseng*,* quinquefolius*, and* salvia* [3–7]. For instance, fourteen nucleotide sequences have been deposited in the NCBI GenBank database until August 2015; among these sequences, six are related to the biosynthetic pathway of triterpenoid saponins in* P. tenuifolia*. The lack of sequence data has limited extensive and intensive studies on* P. tenuifolia*; nevertheless, genomic research related to this medicinal plant is feasible. With the medicinal and economic importance of* P. tenuifolia*, genomic data sources of this plant species should be investigated to discover genes and develop further functional studies.

DNA sequencing technology was developed by Frederick Sanger in 1977. The “first-generation sequencing” was laborious and costly. After years of improvement, next-generation sequencing (NGS) has been developed in terms of speed and accuracy with reduced cost and manpower. Typical examples of NGS include SOLiD/Ion Torrent PGM from Life Sciences, Genome Analyzer/HiSeq 2000/MiSeq from Illumina, and GS FLX Titanium/GS Junior from Roche [8]. Among these techniques, Illumina HiSeq 2000 yields the largest output and entails the lowest reagent cost; this technique has been commonly used in deep sequencing of model and nonmodel organisms [9]. For example, Illumina HiSeq 2000 has been successfully applied in a wide range of species, including model plants (*Arabidopsis*, rice, tomato) [10–12], and crops with large genomes, including field pea, castor, and* Sorghum propinquum* [13–15]. This technique could promote studies on not only the genomics of these plants but also the biosynthetic pathways of the main active ingredients.

Triterpenoid saponins, xanthones, and oligosaccharide esters are currently considered as the main active ingredients of* P. tenuifolia* [16]. However, small amounts of secondary metabolites, such as isoquinoline alkaloids, lignins, and flavonols, are present in* P. tenuifolia*. Indeed, Illumina sequencing should be applied to determine the number of enzymes implicated in the biosynthetic pathway of secondary metabolites in* P. tenuifolia*. To date, the biosynthetic pathway of triterpenoid saponins, particularly pentacyclic triterpenoid saponins in* P. tenuifolia*, has been extensively studied [2]. The formation of a primary cucurbitane skeleton via the isoprenoid pathway is before the cyclization of 2,3-oxidosqualene of terpenoid biosynthesis in plant. Mevalonate acid (MVA) pathway located in cytoplasm and endoplasmic reticulum, and 2-C-methyl-d-erythritol-4-phosphate/1-deoxy-d-xylulose-5-phosphate (MEP/DOXP) pathway located in plasmid, which are likely to participate in the formation of a presenegenin skeleton in* P. tenuifolia*. These two pathways are present in the different cellular parts of green plants [17, 18]. In addition, phenylpropanoid biosynthetic pathways in* P. tenuifolia* are yet to be reported. Phenylalanine is an end product of the shikimate pathway, in which the aromatic amino acids tyrosine and tryptophan are also produced [19]. With phenylalanine as a precursor, lignin biosynthesis proceeds via a series of side-chain modifications, ring hydroxylations, and* O*-methylations to yield lignin monomers [20]. In this pathway, other small molecules, such as flavonoids, coumarins, hydroxycinnamic acid conjugates, and lignans, are also produced. Many of these molecules could be or have been the main focus of studies [21]. These two metabolic pathways are mainly found in* P. tenuifolia*. Therefore, whole transcripts for complete gene expression profiling should be identified by transcriptome sequencing because of limited genomic sources and information regarding the biosynthetic pathways of secondary metabolites in* P. tenuifolia*. It will support further studies on these two metabolic pathways by using high-throughput Illumina deep-sequencing techniques.

In this study, a cDNA library was constructed to obtain detailed and general data from* P. tenuifolia* by using a high-throughput Illumina deep-sequencing technique. We could determine genes encoding the enzymes involved in whole biosynthetic pathways of triterpenoid saponins, phenylpropanoids, and other secondary metabolites, and results could promote further analysis. Furthermore, our results could provide direct experimental data of* P. tenuifolia *not only for guiding genetic breeding but also for subsequent triterpenoid biosynthesis cloning and functional verification of key genes. Therefore, this transcriptome sequencing may help improve future genetics and genomics studies on molecular mechanisms associated with the chemical compositions of* P. tenuifolia*. Eventually, we can control the quality of* P. tenuifolia* from germplasm sources and thus provide important theoretical and practical significance.

## 2. Materials and Methods

### 2.1. Plant Materials

Fresh roots of one-, two-, and three-year-old* P. tenuifolia *were sampled and collected in Xinjiang County, Shanxi Province, China. Another batch of two-year-old fresh roots of* P. tenuifolia *was collected in Anguo City, Hebei Province. All of the samples were intact and healthy. The samples were snap-frozen in liquid nitrogen and stored at −80°C before use.

### 2.2. RNA Extraction, cDNA Library Construction, and Illumina Sequencing

According to the manufacturer's protocol, a plant RNA isolation kit (AutoLab) was used to extract the total RNA from fresh tissues and then purified by an RNeasy MiniElute cleanup kit (Qiagen). In brief, mRNA was purified with the combination of oligo-dT-containing beads and poly-A-containing mRNA and then used methods of chemicals and high temperature to fragmentate the mRNA and get fragments of 150 bp. The next step was to synthesize the double-stranded cDNA and repair the DNA fragments with protruding terminal through the function of 3′-5′ exonuclease and polymerase. In order to guarantee DNA fragments and adapters can combine with “A” and “T” complementary pair connection and prevent the inserted DNA fragments connecting with each other, single-base “A” was imported to the 3′ end of the blunt DNA and single-base “T” was imported to the 3′ end of the adapters. Adapters of the tag-containing and the DNA fragments were incubated and connected by the function of ligase. The free adapters and nonadapters DNA fragments were purified by using AMPureXP beads. DNA fragments with adapters were enriched selectively by PCR, and the PCR amplification with fewer number of cycles was operated in order to avoid errors in the library.

The methods of quantitative of library were Pico green and fluorescence spectrophotometer (Quantifluor-ST fluorometer, Promega, E6090; Quant-iT PicoGreen dsDNA Assay Kit, Invitrogen, P7589), and the quality control of the enrichment of the PCR fragments and validation of the size and distribution of DNA fragments in the library were conducted using Agilent 2100 (Agilent 2100 Bioanalyzer, Agilent, 2100; Agilent High Sensitivity DNA Kit, Agilent, 5067-4626). The samples mixed after homogenization to 10 nM, then diluted gradually and quantitated to 4~5 pM for Illumina sequencing to construct the multiplexed DNA libraries. Illumina sequencing was conducted with Illumina HiSeqTM2000 in CapitalBio, Ltd., Beijing, China.

### 2.3. De Novo Assembly and Functional Annotation

Sequencing of the original data sequence through quality analysis after removal of low quality and subsequences gets the sequences available for subsequent analysis. A Perl program was written to remove reads with adapters, the reads with the average quality of less than Q20 for its 3′ end to the 5′ end, the reads with the final length of less sequence than 50 bp, and the reads with the uncertainty bases. High-quality reads were used for de novo assembly by the Trinity software to construct unique consensus sequences. Trimmed solexa transcriptome reads were mapped onto unique consensus sequences by using Bowtie [22] (Bowtie parameters: -v3 -all -best -strata). The functional annotation of unigenes was compared with National Center for Biotechnology Information (NCBI), nonredundant protein database (Nr), nonredundant nucleotide databases (Nt), UniProt/Swiss-Prot, Kyoto Encyclopedia of Genes and Genomes (KEGG), Clusters of Orthologous Groups (COG), Gene Ontology (GO), and Interpro databases with Basic Local Alignment Search Tool (BLAST, http://www.ncbi.nlm.nih.gov/). Unigenes were identified by comparing sequence similarity against SWISS-PROT (SWISS-PROT downloaded from European Bioinformatics Institute; ftp://ftp.ebi.ac.uk/pub/databases/swissprot/), COG [23, 24], and KEGG database [25] with BLAST at *E* values ≤1*e* − 10. The KO information retrieved from blast results by a Perl script and the pathways between databases and unigenes were established. InterProScan [26] was used to annotate the InterPro domains. Then, the functional assignments of InterPro domains were mapped onto GO and the GO classification and tree were performed by WEGO [27].

## 3. Results

### 3.1. Sequence Assembly

**Figure 1:**
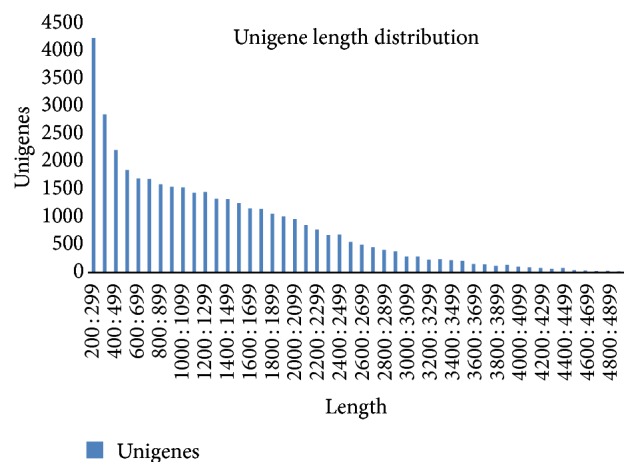
Length distribution of unigenes.

The RNA extracted from all the samples was mixed for Illmina sequencing in order to get the whole range of transcript diversity. In total, 58.88 million raw reads and 11.77 gigabase pairs were sequenced with an average GC content of 43.9%, *Q* ≥ 20, and no ambiguous “N” (Table S1, in Supplementary Material available online at http://dx.doi.org/10.1155/2015/782635). A total of 55,432,632 high-quality reads were assembled, and 316,703 contigs and 145,857 transcripts with the N50 values were 1,636 bp. All the transcripts were subjected to cluster and assembly analyses, yielding 39,625 unigenes with a mean length of 1,378 bp and the N50 values were 1,971 bp (Figure 1). The assembly statistics of contigs, transcripts, and unigenes were shown in Table S2.

### 3.2. Functional Annotation and Classification

In the present library, approximately 67.2% of the unigenes were annotated by BLASTX and BLASTN search with a threshold of 10^−5^ against seven public databases, including Nr, Nt, UniProt/Swiss-Prot, KEGG, COG, GO, and Interpro databases (Table 1).

#### 3.2.1. GO Annotation

**Figure 2:**
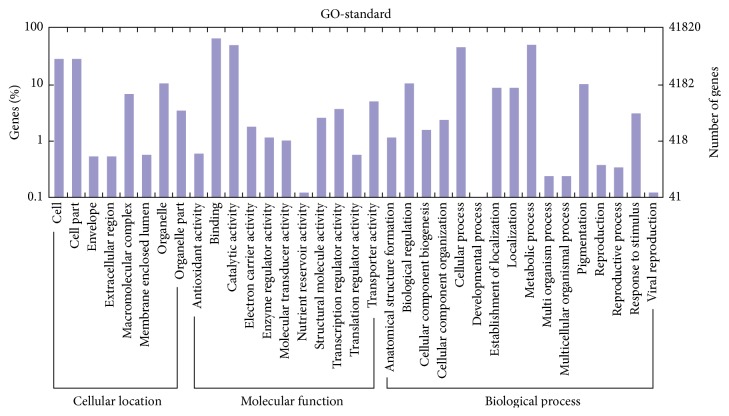
Gene ontology classification assigned to the unigenes.

A total of 41,832 unigenes for GO annotation were divided into three major categories, including cellular location, molecular function, and biological process. Among 34 functional groups with GO assignments, the dominant biochemistry, cell apoptosis, and metabolism were annotated (Figure 2).

In cellular location, eight classifications were clustered by the matched unique sequences and the larger subcategories were “cell” and “cell part.” In terms of molecular function, 11 classifications were categorized, including the represented cellular components, such as “binding” and “catalytic activity.” According to biological processes, 16 classifications were divided, including the represented biological processes, such as “cellular process” and “metabolic process.” However, few genes were assigned to the categories “nutrient reservoir activity” and “viral reproduction,” and no genes were found in the clusters of “developmental process.”

#### 3.2.2. COG Annotation

**Figure 3:**
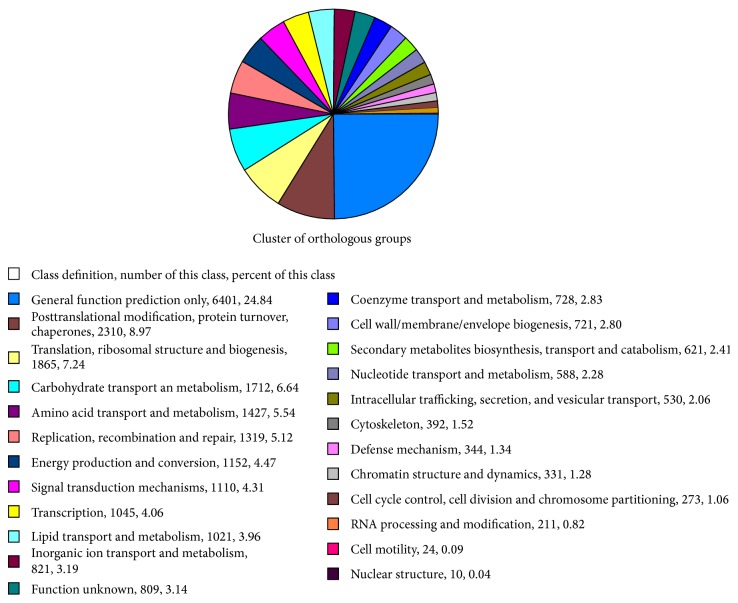
COG classification assigned to the unigenes.

Unigenes for the COG classifications were annotated in order to further evaluate the effectiveness of the annotation process (Figure 3). Class definition, number, and percentage of this class were shown in Figure 3. The proteins in the COG categories were assumed to have the same ancestor protein, and these proteins were also assumed to be paralogs or orthologs. The largest category was general functional prediction only with 24.84%; the second categories were posttranslational modification, protein turnover, and chaperon with 8.97%; and the third category included translation, ribosomal structure, and biogenesis with 7.24%. Cell motility (24, 0.09%) and nuclear structure (10, 0.04%) represented the smallest category.

#### 3.2.3. KEGG Annotation

The KEGG database can be used to categorize gene functions with an emphasis on biochemical pathways. The KEGG database can also be used to systematically analyze inner cell metabolic pathways and functions of gene products. Pathway-based analyses help further determine the biological functions of genes. To gain further insights into the biological pathways in* P. tenuifolia*, we performed a BLASTX search against the KEGG protein database on the assembled unigenes. A total of 72,636 (62.9%) unigenes had blast hits and 42,702 unigenes were assigned to five KEGG biochemical pathways (Table 2) including metabolism (17,217 unigenes), genetic information processing (14,593 unigenes), environmental information (2719 unigenes), cellular processes (3919 unigenes), and human diseases (4254 unigenes). The pathways with the highest unigene representation were spliceosome (ko03041, 1431 unigenes), chromosome (ko03036, 1232 unigenes), and ubiquitin system (ko04121, 1060 unigenes).

**Figure 4:**
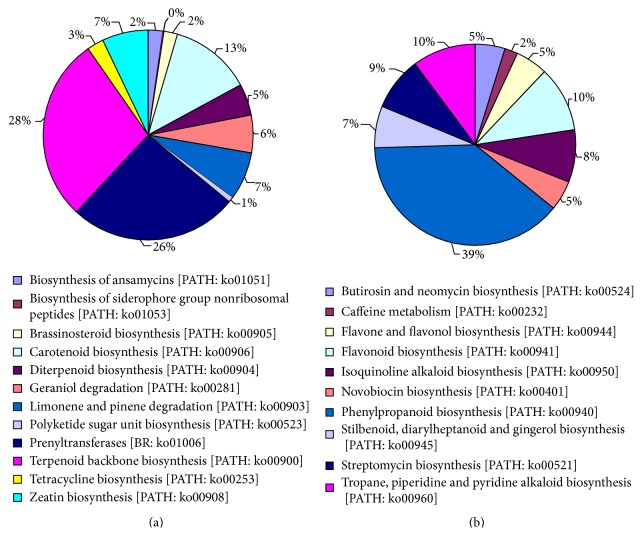
Classifications of unigenes involved in the metabolism of terpenoids and polyketides (a) and biosynthesis of other secondary metabolites (b).

As a Chinese traditional medicinal plant,* P. tenuifolia *undergoes highly active metabolism. The higher expression of metabolism results in primary metabolite biosynthesis, such as “carbohydrate metabolism” (3702 unigenes), “amino acid metabolism” (2168 unigenes), and “energy metabolism” (1762 unigenes). However, the most active ingredients of* P. tenuifolia* were secondary metabolites. Therefore, we listed the classifications of terpenoids, polyketides, and other secondary metabolites (Figure 4) with high expression levels.

Metabolic pathways were involved in the metabolism of terpenoids and polyketides (620 unigenes; Figure 4(a)), including “terpenoid backbone biosynthesis” (176 unigenes, 28%), “carotenoid biosynthesis” (78 unigenes, 13%), “zeatin biosynthesis” (44 unigenes, 7%), “limonene and pinene degradation” (46 unigenes, 7%), “diterpenoid biosynthesis” (30 unigenes, 5%), “tetracycline biosynthesis” (16 unigenes, 3%), “brassinosteroid biosynthesis” (13 unigenes, 2%), and “polyketide sugar unit biosynthesis” (5 unigenes, 1%). The large amount of transcriptomic information may enhance the study of terpenoid biosynthesis in* P. tenuifolia*.

Metabolic pathways were also involved in the biosynthesis of other secondary metabolites (522 unigenes; Figure 4(b)), including “phenylpropanoid biosynthesis” (202 unigenes, 39%), “flavonoid biosynthesis” (55 unigenes, 11%), “tropane, piperidine, and pyridine alkaloid biosynthesis” (53 unigenes, 9%), “streptomycin biosynthesis” (45 unigenes, 9%), “isoquinoline alkaloid biosynthesis” (44 unigenes, 8%), “stilbenoid, diarylheptanoid, and gingerol biosynthesis” (35 unigenes, 7%), “flavone and flavonol biosynthesis” (27 unigenes, 5%), and “butirosin and neomycin biosynthesis” (25 unigenes, 5%). In our sequence dataset, a total of 176 unigenes were found to be potentially related to the biosynthesis of terpenoid biosynthesis including MEP and MVA pathways. In addition, 202 and 55 unigenes were related to the biosyntheses of phenylpropanoid and flavonoid, respectively.

**Figure 5:**
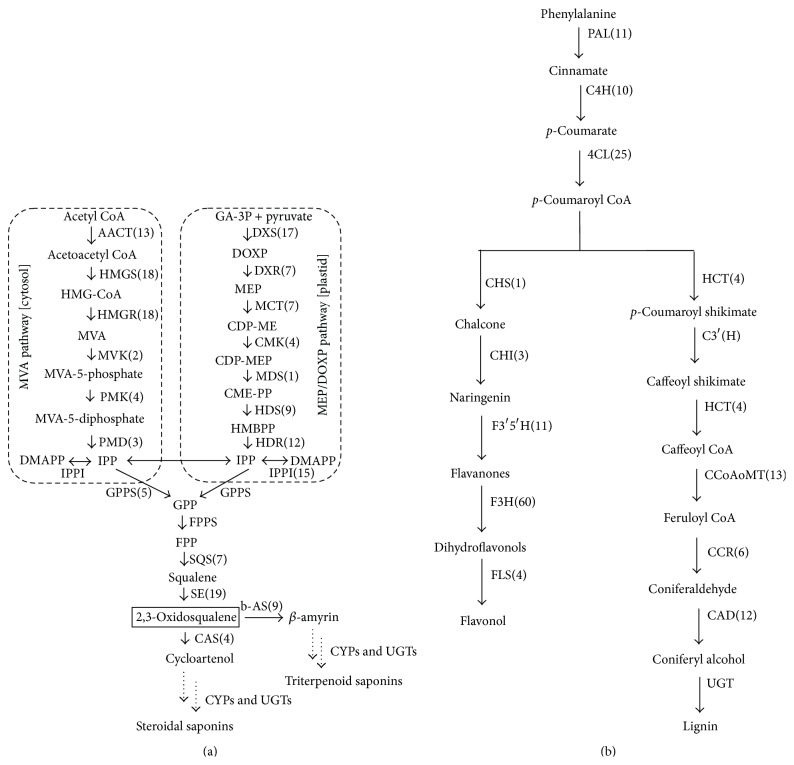
Schematic of the putative biosynthetic pathways of two major classes of active compounds in* P. tenuifolia. *Saponin biosynthesis pathway (a) and phenylpropanoid biosynthesis pathway (b). Enzyme abbreviations: AACT, acetyl CoA C-acetyltransferase or acetoacetyl-CoA thiolase; HMGS, 3-hydroxy-3-methylglutaryl CoA synthase; HMGR, 3-hydroxy-3-methylglutaryl CoA reductase; MVK, mevalonate kinase; PMK, phosphomevalonate kinase; PMD, mevalonate pyrophosphate decarboxylase; DXS, 1-deoxy-d-xylulose-5-phosphate synthase; DXR, 1-deoxy-d-xylulose-5-phosphate reductoisomerase; MCT, 2-C-methyl-erythritol 4-phosphate cytidylytransferase; CMK, 4-(Cytidine 5′-diphospho)-2-C-methyl-d-erythritol kinase; MDS, 2-C-methyl-d-erythritol 2,4-cyclodiphosphate synthase; HDS, 1-hydroxy-2-methyl-butenyl-4-diphosphatesynthase; HDR, isopentenyl pyrophosphate (IPP)/3, 3-dimethylallyl pyrophosphate (DMAPP) synthase; IPPI, isopentenyl pyrophosphate isomerase; GPPS, geranyl diphosphate synthase; FPPS, farnesyl diphosphate synthase; SQS, squalene synthase; SE, squalene epoxidase; CAS, cycloartenol synthase; *β*-AS, *β*-amyrin synthase; DS, dammarenediol synthase; PAL, phenylalanine ammonia lyase; C4H, cinnamate 4-hydroxylase; 4CL, 4-coumarate CoA ligase; CHS, chalcone synthase; CHI, chalcone isomerase; F3′5′H, flavonoid 3′,5′-hydroxylase; F3H, flavanone 3-hydroxylase; FLS, flavonol synthase; HCT, hydroxycinnamoyl-transferase; C3′H,* p*-coumaroyl shikimate 3′ hydroxylase; CCoAoMT, caffeoyl CoA 3-*O*-methyltransferase; CCR, cinnanoyl-CoA reductase; CAD, cinnamyl alcohol dehydrogenase; CYPs, cytochrome P450s; UGTs, glycosyltransferases.

*(1) Characterization and Expression Analysis of the Genes Involved in the Biosynthetic Pathway of Putative Terpenoid Backbone*. Dimethylallyl pyrophosphate (DMAPP) and isopentenyl pyrophosphate (IPP) are recognized as a precursor of the biosynthesis of terpenoid saponins in green plants and the compounds of “activity of isoprene”* in vivo*. DMAPP and IPP are alletic isomers, which are synthesized by MVA and MEP biosynthesis pathways mentioned above [28]. The MVA pathway starts with acetyl-CoA which is synthesized through the triterpene derivatives captured CO_2_ in cytoplasm and the MEP pathway starts with pyruvate and glyceraldehydes-3-phosphate in plastid [29]. In these databases, the biosynthetic pathways of MVA and MEP were identified (Figure 5(a)). In most cases, two or more unique sequences were labeled as the same enzyme and these unique sequences could represent single gene or different fragments of gene.

The process of MVA pathway was as follows. Firstly, two molecules of acetyl-CoA were catalyzed into acetoacetyl CoA by AACT (EC 2.3.1.9, 13 unigenes) and then catalyzed into 3-hydroxy-3-methylglutaryl-CoA (HMG-CoA) by HMGS (EC 2.3.3.10, 18 unigenes). MVA was formulated by HMGR (EC 1.1.1.34, 18 unigenes) and then MVK (EC 2.7.1.36, 2 unigenes) catalyzed MVA into MVA-5-Phosphat. Next, IPP was synthesized by PMK (EC 2.7.4.2, 4 unigenes) and PMD (EC 4.1.1.33, 3 unigenes). The process of MEP pathway was as follows. Firstly, pyruvate and glyceraldehydes-3-phosphate were converted to 1-deoxy-d-xylulose-5-phosphate (DXOP) by DXS (EC 2.2.1.7, 17 unigenes) and then catalyzed into MEP by DXR (EC 1.1.1.267, 7 unigenes). Next, MCT (EC 2.7.7.60, 7 unigenes) and CMK (EC 2.7.1.148, 4 unigenes) catalyzed the MEP to 4-dicytidine triphosphate-2-methyl-d-erythritol-2-phosphate (CDP-MEP). Lastly, IPP was synthesized by MDS (EC 4.6.1.12, 1 unigene), HDS (EC 1.17.7.1, 9 unigenes), and HDR (EC 1.17.1.2, 12 unigenes). The next step was the synthesis of squalene; a series of enzymes were involved, such as IPPI (EC 5.3.3.2, 15 unigenes) and SQS (EC 2.5.1.21, 7 unigenes). However, GPPS (EC 2.5.1.29) and FPPS (EC 2.5.1.10) involved in terpenoid biosynthesis were not found from the transcriptome results maybe because some assembly cDNAs were not of full length. Squalene was catalyzed into 2,3-oxidosqualene by SE (EC 1.14.99.7, 19 unigenes). In many plants 2,3-oxisqualene cyclization was a very important step in the biosynthesis of terpenes, because it was a branch point of the synthesis of triterpenoid saponin, sterol, and other terpenes [30]. We have found that two types of OSC genes of* P. tenuifolia *are present in the dataset; these types are cycloartenol synthase (16 unigenes) and beta-amyrin synthase (54 unigenes), respectively. Moreover, except for the FPPS, another five nucleotide sequences (SQS, SE, CAS (2), cycloartenol synthase, and beta-amyrin) related to the triterpenoid saponins biosynthesis and reported in NCBI were found in our sequencing data.

*(2) Characterization and Expression Analysis of the Genes Involved in the Putative Phenylpropanoid Biosynthesis Pathway*. Approximately 814 unigenes, encoding 16 enzymes, were found in the phenylpropanoid biosynthesis pathway (Figure 5(b)). But flavonoids and lignins were not main compound of phenylpropanoids and researchers were rarely mentioned in* P. tenuifolia*. phenylpropanoid biosynthesis pathway starts with converting phenylalanine to Cinnamate by PAL (EC 4.3.1.24, 11 unigenes) and then Cinnamate converts to* p*-coumaryol CoA by the enzymes of C4H (EC 1.14.13.11, 10 unigenes) and 4CL (EC 6.2.1.12, 25 unigenes).

The flavonoid pathway began with the synthesis of chalcone which was catalyzed by CHS (EC 2.3.1.74, 1 unigenes) and then catalyzed to naringenin by CHI (EC 5.5.1.6, 3 unigenes). Dihydrotricetin was synthesized by F3′5′H (EC 1.14.13.88, 11 unigenes). F3H (EC 1.14.11.9, 60 unigenes) catalyzed these flavanones to dihydroflavonols. The last step was dihydroflavonols converted to flavonols by FLS (EC 1.14.11.23, 4 unigenes).

The lignin pathway began with the synthesis of* p*-coumaroyl shikimate, which was catalyzed by HCT (EC 2.3.1.133, 4 unigenes). Then, C3′H (EC 1.14.13.36) catalyzed the conversion of* p*-coumaroyl shikimate into caffeoyl shikimate. Caffeoyl shikimate was converted by hydroxycinnamoyl-transferase to produce caffeoyl CoA. CCoAoMT (EC 2.1.1.104) could convert these caffeoyl CoA to feruloyl CoA. Then, CCR (EC 1.2.1.44, 6 unigenes) catalyzed the feruloyl CoA into coniferaldehyde. Finally, coniferaldehyde was converted by CAD (EC 1.1.1.195, 12 unigenes) to produce coniferyl alcohol. Similar to the biosynthesis of the terpenoid backbone, C3′H was not found from the transcriptome results.

### 3.3. Simple Sequence Repeat (SSR) Marker Discovery

A total of 6,655 SSRs and 5784 sequences were identified from 39,625 unigenes. Among these sequences, there was more than one SSR for 755 unigene sequences. Mononucleotide accounted for 51.75% and 31.85% for dinucleotide and 14.97% for trinucleotide motify (Table 3). A/T (1539) was the most abundant repeat type, followed by AT/GA (207) and GAA/TCA (69). However, the number of tetranucleotide, pentanucleotide, and hexanucleotide motifs was <2% in the unigene sequences. In this transcriptome database, SSRs did not all exist in the unigenes and the complex SSR motifs were not detected.

## 4. Discussions

The advantages of high-throughput, accuracy, and reproducibility lead transcriptome sequencing to become a powerful technology [31]. Illmina sequencing 2000 has been widely used for deep sequencing for model and nonmodel plants recently with the biggest output and lowest reagent cost. In this study, the application of Illumina HiSeq 2000 resulted in a more comprehensive understanding of genomic information and promoted the study of secondary metabolites biosynthetic pathways of* P. tenuifolia.*

Triterpenoid saponins, especially pentacyclic triterpenoid saponins, are one of the main active ingredients of* P. tenuifolia*, whose biosynthetic pathways are becoming more and more important. The upstream and midstream biosynthetic pathways of triterpenoid saponins have been studied very clearly in the past few years. Many researchers now mainly focus on the downstream biosynthetic pathways, which is also the difficult part of the studies [17]. In the downstream of biosynthesis of natural plant products, hydroxylation of CYP450s and glycosylation of UGTs play important roles in stabilizing the product and altering triterpenoid saponin bioactivity of dammarane-type and oleanane-type aglycones [32]. In this study, 466 and 157 unigenes are annotated as CYP450s and UGTs in the transcriptome of* P. tenuifolia* (data not shown), respectively. However, because of the quantity and complexity of CYP450s and UGTs, the enzymes of the downstream biosynthetic pathway of* P. tenuifolia* are still unknown, which can determine the specific steps in the accumulation of triterpenoid saponins.

CYP450s is a family of enzymes involved in the biosynthesis of lignins, terpenoids, sterols, fatty acids, hormones, pigments, and defense-related phytoalexins [33]. Only two CYP450s involved in triterpene saponin biosynthesis are functionally characterized until now, including CYP88D6 from* Glycyrrhiza uralensis* belonging to the CYP85 family [34] and CYP93E1 from* Glycine max* belonging to the CYP71 family [35]. Recently, several CYP450s and UGTs were identified as candidate genes in many studies of medicinal and nonmedicinal plants. One CYP450 (contig00248) was selected as a candidate gene, which is most likely to be involved in ginsenoside biosynthesis in* Panax quinquefolius* by MeJA-inducible and tissue-specific expression patterns [6]. Moreover, Pn02132 and Pn00158, closely related to CYP88D6 and CYP93E1, were selected as candidate CYP450s involved in triterpene saponin biosynthesis in* P. notoginseng* [5]. Furthermore, ten unigene sequences were identified corresponding to the seven different genes with a high homology to CYP79s, and four unigenes were identified corresponding to the two CYP83 genes in core structure biosynthesis of the glucosinolate metabolism of radish [36]. Our previous study once suggested that a combination of ultraperformance liquid chromatography coupled with electrospray ionization quadrupole time-of-flight mass spectrometry based on metabolomics and gene expression analysis can effectively elucidate the mechanism of biosynthesis of triterpenoid saponin, and three genes of CYP450s (CYP88D6, CYP716B1, and CYP72A1) putatively expressed in biosynthesis pathway of triterpenoid saponin in* P. tenuifolia *were identified by quantitative real-time PCR (qRT-PCR) analysis [2]. The examples mentioned above clarified that most identified CYP450s belonging to the CYP71 and CYP 88 families, and few studies were documented about the other CYP450 families until now.

UGTs are another large multigene family in plants and play an important role in the last step of biosynthesis of triterpenoid saponin. Glycosylation, the transfer of activated saccharides to an aglycone substrate, is the predominant modification in triterpenoid saponins biosynthesis. Regarding CYP450s genes, some UGT genes have also been identified. The genes UGT74M1 and UGT73K1 from* Saponaria vaccaria* [37] and UGT71G1 from* Medicago truncatula* [38] have been previously identified. Flavonoids related to UGTs (UGT78D1, UGT78D2, UGT73C6, and UGT89C1) were also identified in previous studies [39, 40]. Furthermore, four UGTs (contig01001, contig14976, contig15451, and contig16321) were selected as candidate genes in* P. quinquefolius*, which are most likely to be involved in ginsenoside biosynthesis by MeJA-inducible and tissue-specific expression patterns [6]. Pn13895, closely related to UGT71G1, was regarded as a lead candidate UGT, which is responsible for triterpene biosynthesis in* P. notoginseng *[5]. Six putative flavonol glycosides were identified in* Isatis indigotica *[41]. Thirteen unigenes were identified as UGT74s, including UGT74B1, UGT74C1, UGT74F1, and UGT74F2, in the core structure biosynthesis of the glucosinolate metabolism of radish [36]. In our previous study mentioned above [2], three genes of UGTs (UGT74B1, UGT73B2, and UGT73C6) putatively expressed in triterpenoid saponin biosynthetic pathways were also identified by qRT-PCR in* P. tenuifolia*. The examples mentioned above clarified that most identified UGTs belonged to the UGT74 and UGT73 families, and few studies have been documented about the other UGT families until now.

In other words, the genes identified in CYP450s and UGTs add little information to the knowledge of downstream triterpenoid saponin biosynthesis and thus we will continue with our in-depth studies. The large number of CYP450 and UGTs candidates could provide not only a potential gene pool for the identification of special CYP450s and UGTs involved in triterpenoid biosynthesis in* P. tenuifolia* but also a convenient method to characterize the roles in triterpenoid biosynthesis in future studies.

## 5. Conclusion

RP, one of the predominant Chinese medical plants, contains various medicinal ingredients with the elicits tonic, sedative, antipsychotic, expectorant, and other pharmacological effects. In this study, a large-scale unigene investigation of* P. tenuifolia* was performed by Illumina sequencing. Our results showed that many transcripts encoded by putative genes are involved in the biosynthesis of triterpene saponins and phenylpropanoid. The data we obtained presented the most abundant genomic resource and provided comprehensive information on gene discovery, transcriptome profiling, transcriptional regulation, and molecular markers of* P. tenuifolia*. This study will improve the production of active compounds through marker-assisted breeding or genetic engineering for* P. tenuifolia*, as well as other medicinal plants in the Polygalaceae family. However, many candidate CYP450s and UGTs are likely involved downstream of the biosynthetic pathway of triterpene saponins, but these candidates should be further investigated.

## Supplementary Material

TABLE S1: Length distribution of raw data. The 58.88 million raw reads and 11.77 gigabase pairs were sequenced with an average GC content of 43.9%, *Q*≥ 20 and no ambiguous “N” in *P. tenuifolia*. The length of reads is 101 bp. Q20 and Q30 mean that the percentages of the bases, which quality values ≥ 20 or 30.
TABLE S2: Length distribution of assembled contigs, transcripts, and unigenes. A total of 55,432,632 high-quality reads were assembled, 316,703 contigs, 145,857 transcripts and 39,625 unigenes were obtained with the N50 values was 522 bp, 1,636 bp and 1971 bp. The max length of contigs, transcripts and unigenes were 16,007 bp, 16,008 bp and 16,008 bp. The average length of contigs, transcripts and unigenes were 310.76 bp, 970 bp and 1,378 bp. The number of >N50 reads of contigs, transcripts and unigenes were 38,638, 27,911 and 9,484.

## Figures and Tables

**Table 1:** Functional annotation of *P. tenuifolia*.

Database	Total		*E* cutoff	Database version
	Number of annotated	Percent (%)		
Total unigenes	115,477			
Nt	69,211	59.9	1.00*e* − 05	201301
Nr	77,599	67.2	1.00*e* − 05	201301
Swissprot	54,582	47.3	1.00*e* − 10	201301
COG	30,548	26.5	1.00*e* − 10	No version
KEGG	72,636	62.9	1.00*e* − 10	Release58
Interpro	49,873	43.19	Interproscan 4.8	v36
GO	41,823	36.22		

**Table 2:** Pathway classification of *P. tenuifolia*.

Category	Pathway	Count
Metabolism	Carbohydrate metabolism	3702
	Amino acid metabolism	2618
	Enzyme families	1781
	Energy metabolism	1762
	Lipid metabolism	1610
	Glycan biosynthesis and metabolism	1390
	Nucleotide metabolism	1055
	Metabolism of cofactors and vitamins	879
	Metabolism of other amino acids	716
	Metabolism of terpenoids and polyketides	620
	Xenobiotics biodegradation and metabolism	562
	Biosynthesis of other secondary metabolites	522

Genetic information processing	Folding, sorting, and degradation	4312
	Translation	4029
	Transcription	3191
	Replication and repair	3061

Environmental information processing	Signal transduction	2050
	Membrane transport	342
	Signaling molecules and interaction	327

Cellular processes	Cell growth and death	1540
	Transport and catabolism	1399
	Cell motility	507
	Cell communication	473

Human diseases	Neurodegenerative diseases	1398
	Infectious diseases	1371
	Cancers	1146
	Immune system diseases	236
	Cardiovascular diseases	103

**Table 3:** Frequency of identified SSR motifs in *P. tenuifolia*.

Motif length	Repeat numbers							Total	%
	5	6	7	8	9	10	>10		
Mononucleotide	—	—	—	—	—	1554	1890	3444	51.75
Dinucleotide	—	638	374	333	333	317	125	2120	31.85
Trinucleotide	646	218	117	10	1	2	2	996	14.97
Tetranucleotide	64	12	0	0	0	0	0	76	1.14
Pentanucleotide	2	1	0	0	0	0	0	3	0.045
Hexanucleotide	12	2	2	0	0	0	0	16	0.24
Total	724	871	493	343	334	1873	2017	6655	
%	10.87	13.09	7.41	5.15	5.02	4.79	1.91		
