# Supplementary material for: Analysis of Polygala tenuifolia Transcriptome and Description of Secondary Metabolite Biosynthetic Pathways by Illumina Sequencing
Source: Int J Genomics. 2015 Oct 12;2015:782635. doi: 10.1155/2015/782635 (PMC4620389; doi:10.1155/2015/782635)
Supplement: Supplementary file 1 — TABLE S1: Length distribution of raw data. The 58.88 million raw reads and 11.77 gigabase pairs were sequenced with an average GC content of 43.9%, Q≥ 20 and no ambiguous “N” in P. tenuifolia. The length of reads is 101 bp. Q20 and Q30 mean that the percentages of the bases, which quality values ≥ 20 or 30. TABLE S2: Length distribution of assembled contigs, transcripts, and unigenes. A total of 55,432,632 high-quality reads were assembled, 316,703 contigs, 145,857 transcripts and 39,625 unigenes were obtained with the N50 values was 522 bp, 1,636 bp and 1971 bp. The max length of contigs, transcripts and unigenes were 16,007 bp, 16,008 bp and 16,008 bp. The average length of contigs, transcripts and unigenes were 310.76 bp, 970 bp and 1,378 bp. The number of >N50 reads of contigs, transcripts and unigenes were 38,638, 27,911 and 9,484. [file 782635.f1.pdf]

TABLE S1. Length distribution of raw data

**Raw Reads**	**Raw Data(bp)**	**Q20(%)**	**Q30(%)**	**GC(%)**	**Reads Len.(bp)**
58,881,563	11,776,3112,600	97.52	93.11	43.90	101

TABLE S2. Length distribution of assembled contigs, transcripts, and unigenes

	**Total Length (bp)**	**Sequence No.**	**Max Length (bp)**	**Average Length (bp)**	**N50**	**>N50 Reads No.**
Contigs	98,418,921	316,703	16,007	310.76	522	38,638
Transcripts	141,535,320	145,857	16,008	970	1,636	27,911
Unigenes	54,594,810	39,625	16,008	1,378	1,971	9,484
